# Pan-cancer analysis reveals the relationship between RCSD1 immune infiltration and clinical prognosis in human tumors

**DOI:** 10.3389/fimmu.2022.1008778

**Published:** 2022-10-13

**Authors:** Han Qiao, Hong Yin, Yan Feng, Huaping Tang

**Affiliations:** ^1^ Department of Respiratory Medicine, Qingdao Municipal Hospital, Qingdao University, Qingdao, China; ^2^ Department of Thoracic Surgery, Qingdao Municipal Hospital, Qingdao, China

**Keywords:** RCSD1, pan-cancer, prognosis, immune infiltration, biomarker

## Abstract

**Background:**

RCSD1 is a cytoskeletal regulator that has been confirmed to undergo genetic mutations in hematological tumors, but the mechanisms of RCSD1 in pan-cancer and its impact on patient prognosis have not been studied.

**Methods:**

Using TCGA, GEPIA, UALCAN, Kaplan-Meier plotters, Linkedomics, String, cBioPortal, TISIDB, TCIA and TIMER database methods, we investigated the expression of RCSD1 in human tumors and its relationship to clinical prognosis, functional analysis of co-expression networks, mutation status, and immune infiltration in cancers, especially lung adenocarcinoma (LUAD) and lung squamous cell carcinoma (LUSC).

**Results:**

The expression of RCSD1 is low in most tumors compared with normal tissues, and its high expression is associated with good patient survival. The RCSD1 co-expression network is mainly involved in the regulation of immune response. In human cancer, RCSD1 plays an important role in the tumor microenvironment (TME) and is significantly associated with the expression of immune infiltrating cells (TIL) in lung cancer.

**Conclusions:**

As a prognostic biomarker of generalized cancer, RCSD1 is associated with immune infiltration.

## Introduction

Worldwide, tumor is a major factor in human death (1). Since most cancers are already advanced at the time of diagnosis, conventional surgery and chemoradiation do not achieve ideal therapeutic outcomes (2).Tumor immunotherapy has become a new means of cancer treatment, and has made important progress in recent years (3). To find new potential tumor therapeutic targets, bioinformatics analysis using public information databases is a more efficient approach (4, 5). Through the pan-cancer analysis of the genes, we have identified novel potential tumor immunotherapy targets that may improve patient survival in the future.

The RCSD1 (RCSD domain protein 1) gene encodes the protein kinase substrate CAPZIP (CAPZ interacting protein), which is mainly found in immune cells and muscle cells and can interact specifically with CAPZ (6, 7). Under certain conditions, CAPZIP phosphorylation activation plays an important role in the assembly of the cytoskeleton (7, 8). Previous studies have stated clearly that the gene fusions of RCSD1 can occur in leukemias (9, 10). Genetic alterations in RCSD1 result in altered cellular function affecting cytoskeletal regulation, which may be a significant procedure in leukemogenesis. However, the impact of RCSD1 on other human malignancies is unclear yet.

In our study, we used a variety of databases to analyze RCSD1 expression, prognostic value, molecular functional networks, genetic alterations, and potential relationships with tumor immune microenvironment in pan-carcinomas. In addition, we explored the relationship between RCSD1 and clinical case parameters of lung cancer, immune cells, and tumor immunotherapy. The purpose of this study was to explore the role of RCSD1 in human cancer, thereby providing insights into new anti-tumor strategies.

## Methods

### TCGA

TCGA (https://portal.gdc.cancer.gov/), a web sites that incorporate clinical data, genomic variation, and other data for cancers (tumors including subtypes) are a major source of data for cancer researchers. RNA expression profile data of lung cancer patients were downloaded from the TCGA database. (Data from a total of 594 LUAD patients, containing 535 tumor cases and 59 normal cases, were collected, and relevant clinical data was obtained for 522 cases. Then data from 551 LUSC patients, including 502 tumor cases and 49 normal cases, were also received, and the related clinical data were obtained for 504 cases.) Then, we detected the proportion of immune infiltrating cells in different expression groups of RCSD1 in the TCGA dataset using R package “CIBERSORT” (11). The correlation between immune cell infiltration level and RCSD1 expression was also analyzed.

### GEPIA

Gene Expression Profiling Interactive Analysis (GEPIA) is an RNA sequencing data platform (http://gepia.cancer-pku.cn/) including 9736 tumor tissues and 8587 normal tissues from the TCGA and GTEx databases (12). Click on the “Box Plots” module in the GEPIA database to retrieve the difference of RCSD1 gene expression in various tumors and normal tissues. Meanwhile, we searched its effect on patient outcomes [disease-free survival (DFS) and overall survival (OS)] in the “Survival” module, and explored the relationship between RCSD1 and various pathological stages in the “Stage Plot” module. Using GEPIA, we identified 100 genes associated with RCSD1. In addition, we explored the relationship between RCSD1 and immune cell biomarkers.

### TIMER

TIMER web server (https://cistrome.shinyapps.io/timer/) is a website for comprehensive analysis of gene expression and tumor-infiltrating immune cells (TIICs) in TCGA cancers (11, 13, 14). We used the “Exploration” module in the TIMER database, clicking on “Gene_DE”, and entered “RCSD1” to retrieve the differences in RCSD1 expression between tumor tissues and normal tissues, analyzed the correlation between RCSD1 and its co-expressed genes. Then we assessed the correlation between RCSD1 and molecular markers of tumor immune infiltrating cells and immune cells. The association between RCSD1 copy number variation (CNV) and immune cell infiltration was then investigated, and the effect of RCSD1 and immune cell expression on patient survival was investigated using Kaplan-Meier curves.

### UALCAN

UALCAN database (http://ualcan.path.uab.edu/index.html) is available for online analysis of differential gene expression in cancer and normal tissue from the TCGA RNA sequencing data and clinical data of 31 malignancies (15, 16). We downloaded the expression of total and phosphorlated proteins of RCSD1 in pan-cancer from the “CPTAC module” in the UALCAN database. In addition, we examined the correlation between clinical data and RCSD1 gene expression.

### Kaplan-Meier plotter

Kaplan-Meier plotter (http://kmplot.com/analysis/) (17) is an open portal tool for prognostic analysis. To determine the relationship between RCSD1 expression and pan-cancer patient outcomes (Relapse Free Survival and Overall Survival rates), we used the Kaplan-Meier plotter database.

### TISIDB

TISIDB (http://cis.Hku.hk/TISIDB/) is a powerful website that contains a large amount of tumor immunity-related data and facilitates a comprehensive study of tumor-immune interactions (18). Here, we used the “Subtype” module to analyze the relationship of RCSD1 expression with pan-cancer immune subtypes and immune molecular subtypes, searched the correlation of RCSD1 and six immune characteristics (lymphocytes, immunomodulators, chemokines) in lung cancer.

### cBioPortal database

The cBioPortal for Cancer Genomics (https://www.cbioportal.org) is an open-source resource for interactive exploration of multidimensional cancer genomics datasets (19, 20). In this study, the “Mutations” module and the “Cancer types summary” module were used to determine the mutation status and site of RCSD1 in tumors, explore the relationship between the RCSD1 gene mutation and DFS, DSS (disease-specific survival), PFS (progression-free survival) and OS in COAD (Colon adenocarcinoma) and PCPG (Pheochromocytoma and Paraganglioma) patients, analyze the correlation between cancer-infiltrating immune cells and RCSD1 expression.

### String database

The String database (https://string-db.org/) can be searched for the interactions between proteins (21). We change “max number of interactors to show” to “no more than 50 interactors” to gain the top 50 proteins with the interaction with RCSD1, preserving the protein interaction network maps and correlation data for subsequent correlation analysis.

### Gene ontology function and Kyoto encyclopedia of genes and genomes pathway enrichment analysis

In the pan-cancer analysis part, GO analysis was used to explore the biological processes (BP), cell components (CC) and molecular functions (MF) of RCSD1-related genes. The GO analysis describes the possible molecular functions, the cellular environment, and the biological processes associated with the RCSD1 gene product. We studied the potential mechanisms of RCSD1-related genes using KEGG pathway enrichment analysis. Next, the GO and KEGG analysis of lung cancer was performed by the R software package ClusterProfiler.

### Linkedomics

LinkedOmics (http://www.linkedomics.org/login.php) (22) can analyze and compare cancer multi-omics data within and across tumor types. We first select “TCGA_LUAD” in “STEP-1”, then enter “RCSD1”, and finally select the statistical method. The Pearson correlation coefficient was used to identify co-expressed genes for RCSD1, and heatmaps and volcano plots are used to illustrate the results. In addition, we explored the GO analysis and KEGG pathways of RCSD1 and its co-expressed genes, using a gene set enrichment analysis (GSEA).

### The Cancer Immunome Atlas

The Cancer Imaging Archive (TCIA) (http://www.cancerimagingarchive.net) provides the results of a comprehensive immunogenomic analysis from the TCGA and other datasets (23, 24). To investigate the prognostic value of RCSD1 in tumors and its correlation with the immune micro-environment, we downloaded LUAD, LUSC patient data from the TCIA website and analyzed the effect of immunotherapy with high and low RCSD1 expression groups using R software.

## Results

### RCSD1 gene expression in pan-cancer patients

**Figure 1:**
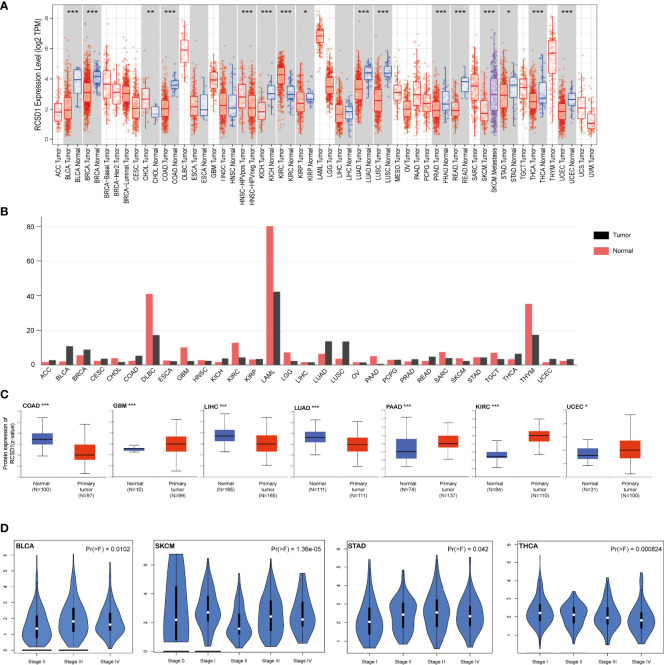
Expression level of the RCSD1 gene in different human tumors and pathological stages. **(A)** Expression status of the RCSD1 gene in different cancer was analyzed by TIMER database. **(B)** RCSD1 expression level in different cancers verified using GEPIA database. **(C)** Expression levels of total RCSD1 protein between normal and primary tissues of COAD, GBM, LIHC, LUAD, PAAD, KIRC and UCEC were extracted and analyzed in the CPTAC dataset. **(D)** The RCSD1 gene expression levels at the major pathological stages of BLCA, SKCM, STAD, and THCA using GEPIA database. *, P <0.05; **, P <0.01; ***, P <0.001.

In this study, we applied the Timer database to analyze the expression levels of RCSD1 in different cancer types. As shown in **Figure 1A**, the expression level of RCSD1 in the tumor tissues of BLCA (Bladder Urothelial Carcinoma), BRCA (Breast invasive carcinoma), COAD, KICH (Kidney Chromophobe), KIRP (Kidney renal papillary cell carcinoma), LUAD, LUSC, PRAD (Prostate adenocarcinoma), READ (Rectum adenocarcinoma), STAD (Stomach adenocarcinoma), THCA (Thyroid carcinoma), and UCEC (Uterine Corpus Endometrial Carcinoma) is lower than the corresponding normal tissues. Only CHOL (Cholangiocarcinoma) and KIRC (Kidney renal clear cell carcinoma) tumors had higher RCSD1 expression than normal tissues. We then used the GEPIA database to find that RCSD1 expression in 15 human tumors was lower than that in normal tissues (**Figure 1B**). Results from the CPTAC datasets showed that the total RCSD1 protein expression was lower in primary COAD, LIHC (Liver hepatocellular carcinoma) and LUAD tissues than in normal tissues. However, the total RCSD1 protein expression was higher in the GBM (Glioblastoma multiforme), PAAD (Pancreatic adenocarcinoma), KIRC, and UCEC primary tissues than in the normal tissues (**Figure 1C**). Meanwhile, BRCA, LUAD and KIRC were selected to study phosphoroprotein expression of RCSD1 in tumors. We can see that the expression of the RCSD1 phosphorylated proteins in LUAD and BRCA was lower than that in the normal tissues (**Figures S1A–L**). In KIRC, RCSD1 phosphoprotein levels at S68, S105, S105S108, and S284 were poorly expressed in tumors and RCSD1 phosphoproteins at S116S120, S216, S267S268, and S298 were highly expressed in tumors(**Figures S1M–T**). Next, we explored whether RCSD1 expression varied across different stages of the same tumor. As we can see, the RCSD1 expression levels differ significantly in the BLCA, SKCM (Skin Cutaneous Melanoma), STAD and THCA tumor pathological stage (**Figure 1D**).

### The prognostic value of RCSD1 in pan-cancer

**Figure 2:**
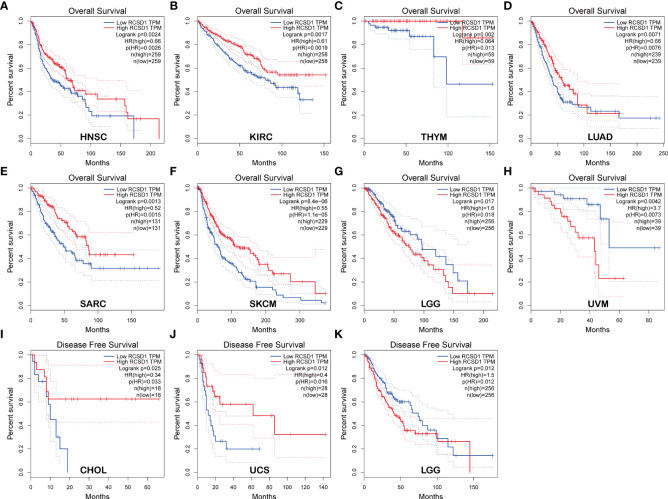
Relationship between RCSD1 expression and patient survival using GEPIA. Relationship between RCSD1 expression and OS of **(A)** HNSC patients, **(B)** KIRC patients, **(C)** THYM patients, **(D)** LUAD patients, **(E)** SARC patients, **(F)** SKCM patients, **(G)** LGG patients, and **(H)** UVM patients. Relationship between RCSD1 expression and RFS of **(I)** CHOL patients, **(J)** UCS patients, and **(K)** LGG patients. OS, Overall Survival; RFS, Disease Free Survival.

**Figure 3:**
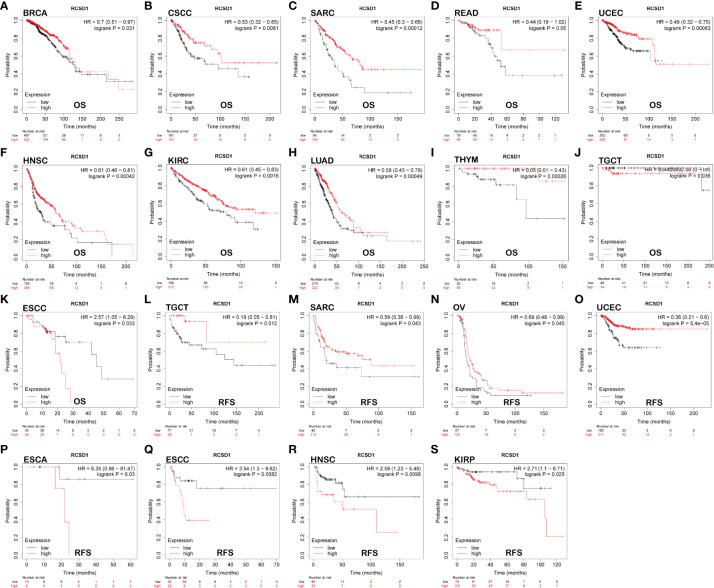
The relationship between the expression of RCSD1 and the prognosis of tumor patients was shown with Kaplan–Meier survival curves. OS of **(A)** BRCA, **(B)** CSCC, **(C)** SARC, **(D)** READ, **(E)** UCEC, **(F)** HNSC, **(G)** KIRC, **(H)** LUAD, **(I)** THYM, **(J)** TGCT and **(K)** ESCC. RFS of **(L)** TGCT, **(M)** SARC, **(N)** OV, **(O)** UCEC, **(P)** ESCA, **(Q)** ESCC, **(R)** HNSC and **(S)** KIRP. OS, Overall Survival; RFS, Relapse Free Survival.

Using GEPIA, we found that low RCSD1 expression was associated with poor OS in HNSC (Head and Neck squamous cell carcinoma), KIRC, THYM, LUAD, SARC (Sarcoma), and SKCM (**Figures 2A–F**); low RCSD1 expression in both LGG (Brain Lower Grade Glioma) and UVM (Uveal Melanoma) was associated with better OS (**Figures 2G, H**). Meanwhile, the analysis results showed that high RCSD1 expression was associated with better RFS in CHOL and UCS (Uterine Carcinosarcoma) patients (**Figures 2I, J**), and instead, high RCSD1 expression was associated with poor RFS in LGG (**Figure 2K**). We then performed the survival analysis using Kaplan-Meier plotter, found that high expression of RCSD1 was associated with better OS of BRCA, CSCC (Cervical squamous cell carcinoma), SARC, READ, UCEC, HNSC, KIRC, LUAD, and THYM (**Figures 3A–I**), while high expression of RCSD1 was correlated with poor OS of TGCT and ESCC (Esophageal squamous cell carcinoma) (**Figures 3J, K**). Meanwhile, poor RFS of TGCT, SARC, OV (Ovarian serous cystadenocarcinoma) and UCEC were correlated with low expression of RCSD1 (**Figures 3L–O**), and poor RFS of ESCC, ESCA (Esophageal carcinoma), HNSC and KIRP was significantly associated with high expression of RCSD1 (**Figures 3P–S**).

### Gene alteration of RCSD1 in pan-cancer

**Figure 4:**
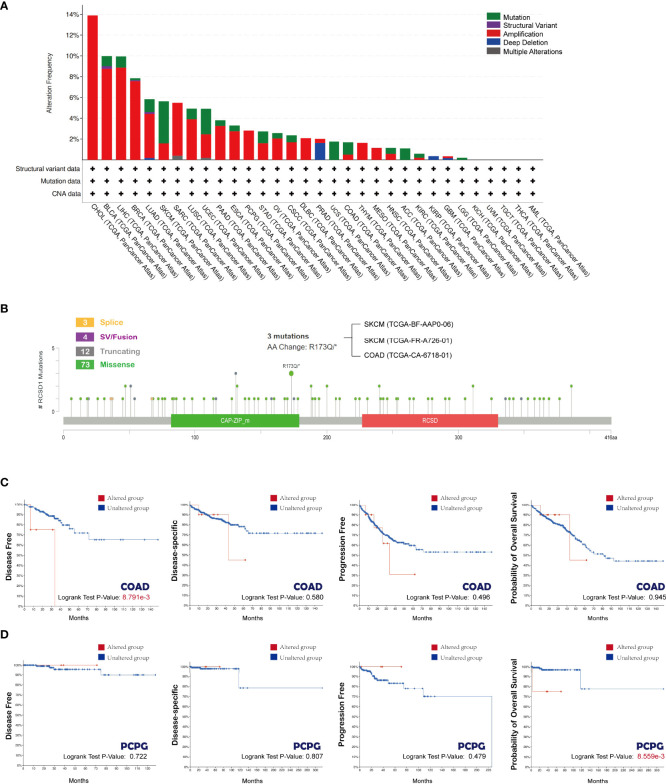
Mutation status of RCSD1 analyzed using the cBioPortal tool. **(A)** Frequency of RCSD1 mutations in different tumors. **(B)** Mutation site of RCSD1. Association of RCSD1 mutations with prognosis of patients with **(C)** COAD and **(D)** PCPG.

Next, we explored the gene mutations of RCSD1 in different tumors. **Figure 4A** shows the type of RCSD1 gene mutation in the 32 cancers from the TCGA database. We can see that the RCSD1 gene can undergo multiple mutations, including mutations, structural variation, amplification, deep deletion, and multiple alterations. Among them, the CHOL patients had the highest frequency of RCSD1 gene change (13.89%), which were all amplified. The frequency of previous genetic alterations in BLCA patients was 9.98%, containing 0.97% mutations, 0.24% structural variant and 8.76% amplifications. The frequency of gene alterations in LUAD patients was 5.83%, including 1.24% mutations, 0.18% structural variant, 4.24% amplification and 0.18% deep deletion. The gene alteration frequency in LUSC patients was 4.93%, including 1.03% mutations and 3.9% amplifications (**Figure 4A**). Then we observed the genetic alteration status of RCSD1 (NM_052862) in different tumor samples of the TCGA cohorts and found that the somatic mutation frequency of RCSD1 reached 0.8%. **Figure 4B** shows the specific mutation case on each domain. R173Q alteration in the tumor domain, which was detected in 2 cases of SKCM and 1 case of COAD, induced a Missense Mutation of the RCSD1 gene (**Figure 4B**). Alterations in the RCSD1 gene may affect the clinical survival outcomes in different types of cancer cases. For example, in the COAD group, the disease-free survival was significantly shortened in the RCSD1 gene alteration group (p=8.791e-3) (**Figure 4C**), and in PCPG group, patients in the unaltered group had better OS than the altered group (p=8.559e-3) (**Figure 4D**).

### Enrichment analysis of RCSD1-related genes in pan-cancer

**Figure 5:**
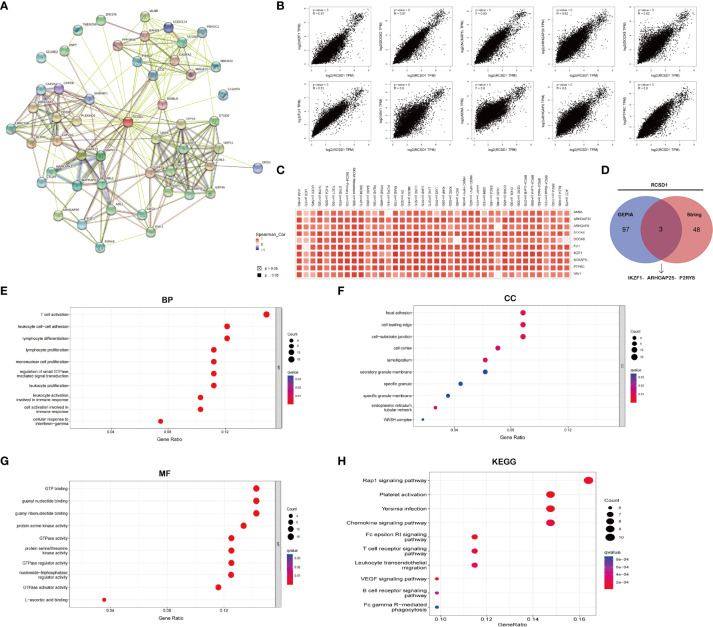
RCSD1 co-expression network. **(A)** RCSD1-binding proteins. **(B)** The correlation of top 10 RCSD1-correlated genes, including AKNA, ARHGAP30, ARHGAP9, DODK2, DODK8, FLI1, IKZF1, NCKAP1L, PTPRC and VAV1. **(C)** Correlation of RCSD1 with the 10 genes in human tumors. **(D)** VENN plots showing the cross-analysis of correlated genes. **(E)** GO_BP analysis. **(F)** GO_CC analysis. **(G)** GO_MF analysis. **(H)** KEGG pathway analysis.

First, we demonstrated the relationship between RCSD1 and 50 proteins that interact with it using String database (**Figure 5A**). Then, the 100 genes with the association with RCSD1 were searched in the GEPIA database. Among them, the 10 genes were all positively associated with RCSD1, namely IKZF1, DOCK2, NCKAP1L, ARHGAP30, DOCK8, FLI1, VAV1, AKNA, ARHGAP9 and PTPRC (**Figure 5B**). The association of RCSD1 with the above 10 genes in different tumors was further analyzed using the Timer database (**Figure 5C**). And then the 50 genes obtained from String and 100 genes downloaded from GEPIA were crossed using Venn plots as the following: P2RY8, ARHGAP25 and IKZF1 (**Figure 5D**). Thereafter, we performed GO and KEGG analysis of these 150 genes. GO analysis is divided into three parts: GO_MF, GO_BP and GO_CC. In the case of GO_BP, the RCSD1-related genes are implicated in the immune response processes, such as “T cell activation”, “Lymphocyte differentiation”, “Mononuclear cell differentiation”, “Lymphocyte proliferation”, and “Mononuclear cell proliferation” (**Figure 5E**). In terms of GO_CC, the RCSD1-related gene product is located at focal adhesion, cell leading edge and cell-substrate junction while performing function (**Figure 5F**). And in the GO_MF analysis, we learned that the RCSD1-related genes mostly have the GTPase-regulator activity, the nucleoside-triphosphatase regulator activity and the GTPase activator activity (**Figure 5G**). KEGG pathway analysis revealed that these 150 genes were mainly associated with cellular immunoregulatory processes such as “Rap1 signaling pathway”, “Plate activation”, “Yersinia infection”, “Chemokine signaling pathway”, “Fc epsilon RI signaling pathway”, and “T cell receptor signaling pathway” (**Figure 5H**).

### Association between RCSD1 expression and pan-cancer immune subtypes and immune molecular subtypes

**Figure 6:**
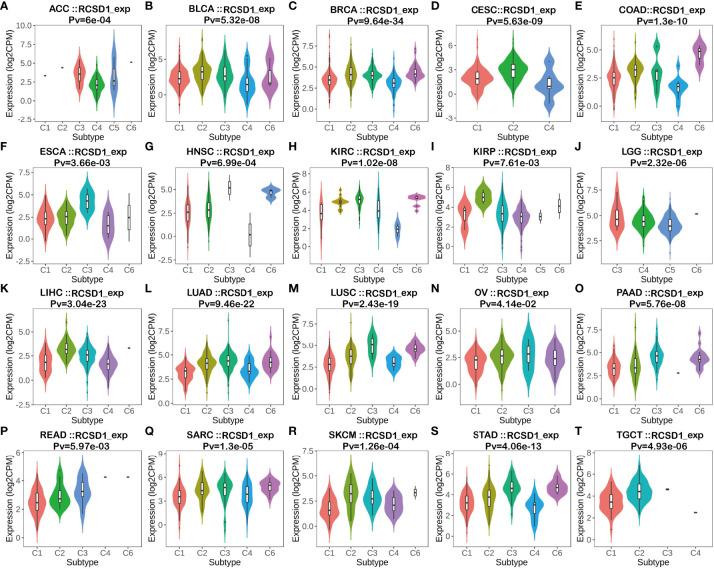
The relationship between RCSD1 expression and pan-cancer immune subtypes. **(A)** in ACC, **(B)** in BLCA, **(C)** in BRCA, **(D)** in CESC, **(E)** in COAD, **(F)** in ESCA, **(G)** in HNSC, **(H)** in KIRC, **(I)** in KIRP, **(J)** in LGG, **(K)** in LIHC, **(L)** in LUAD, **(M)** in LUSC, **(N)** in OV, **(O)** in PAAD, **(P)** in READ, **(Q)** in SARC, **(R)** in SKCM, **(S)** in STAD, **(T)** in TGCT.

From the previous gene enrichment analysis, it is known that RCSD1 may be involved in cellular immune regulation and cellular metabolism-related processes. Therefore, we then analyzed the relationship between RCSD1 expression and immune subtypes by the TISIDB website. The results showed that RCSD1 expression was significantly different among the different immune subtypes (**Figure 6**). Immune subtypes were classified into six types, including C1 (Wound healing), C2 (IFN-gamma dominant), C3 (Inflammatory), C4 (Lymphocyte depleted), C5 (Immunologically quiet) and C6 (TGF-b dominant). Among them, the RCSD1 expression of the C4 subtype was relatively low in ACC, BLCA, BRCA, CESC, COAD, ESCA, KIRP, LIHC and STAD; in both LUAD and LUSC, RCSD1 is highly expressed in C3 and C6 subtype, but it has lower expression in C1 and C4 subtype (**Figures 6L, M**). For different molecular subtypes of cancers, a significant connection with RCSD1 expression existed in ACC, BRCA, COAD, ESCA, HNSC, KIRP, LGG, LIHC, LUSC, OV, PCPG, PRAD, STAD and UCEC (**Figure S2**). In LUSC, secretory subtype expressed the highest RCSD1 and the lowest RCSD1 in primitive subtype (**Figure S2I**).

### Relationship between RCSD1 expression and clinical parameters in lung cancer patients

**Figure 7:**
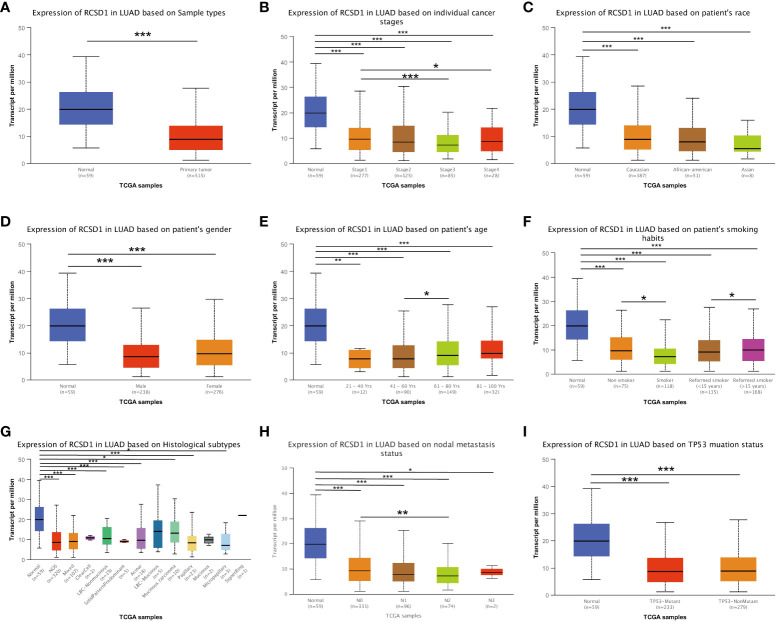
RCSD1 differential expression in LUAD with different clinical subgroups. On **(A)** sample types, **(B)** individual cancer stages, **(C)** patient’s race, **(D)** patient’s gender, **(E)** patient’s age, **(F)** patient’s smoking habits, **(G)** histological subtypes, **(H)** nodal metastasis status and **(I)** TP53 mutation status. *, P < 0.05; **, P < 0.01; ***, P < 0.001.

The relationship between RCSD1 expression and the clinical data of lung cancer patients was analyzed using the UALCAN database. We concluded that, firstly, compared with normal tissues, the expression level of RCSD1 in tumor tissues is lower (**Figure 7A**). RCSD1 is related to individual cancer stage in LUAD patients, RCSD1 expression levels in stage 1 was higher than stage 3 and stage 4 (**Figure 7B**). Secondly, RCSD1 expression was correlated with age in LUAD patients, with higher RCSD1 expression levels in the 61-80 years old group than in the 41-60 years old group (**Figure 7E**). Meanwhile, the expression of RCSD1 was also correlated with the smoking habit of LUAD patients. The non-smoking group expressed higher RCSD1 levels than the smoking group, and the higher RCSD1 level in the reformed smoker (> 15 years) group than the reformed smoker (<15 years) group (**Figure 7F**). Finally, RCSD1 expression was correlated with lymph node metastasis status in LUAD patients, and patients in the N0 group expressed higher levels of RCSD1 than in the N2 group (**Figure 7H**). However, RCSD1 expression was not significantly different with race, sex, histological subtypes, and TP53 mutation status in the LUAD group (**Figures 7C, D, G, I**). Similarly, in LUSC group, the expression of RCSD1 is associated with individual cancer stage, and stage 1 patients’ RCSD1 expression level is higher than that in stage 4 (**Figure S3B**). The expression of RCSD1 was related to the LUSC histological subtypes, the RCSD1 expression levels in the NOS group was higher than in the basaloid group (**Figure S3G**). RCSD1 expression was also associated with TP53 mutation status in LUSC patients, and the RCSD1 expression level was higher in the TP53-NonMutant group than in the TP53-Mutant group (**Figure S3I**).

### Co-expression network and enrichment analysis of RCSD1 in lung cancer

**Figure 8:**
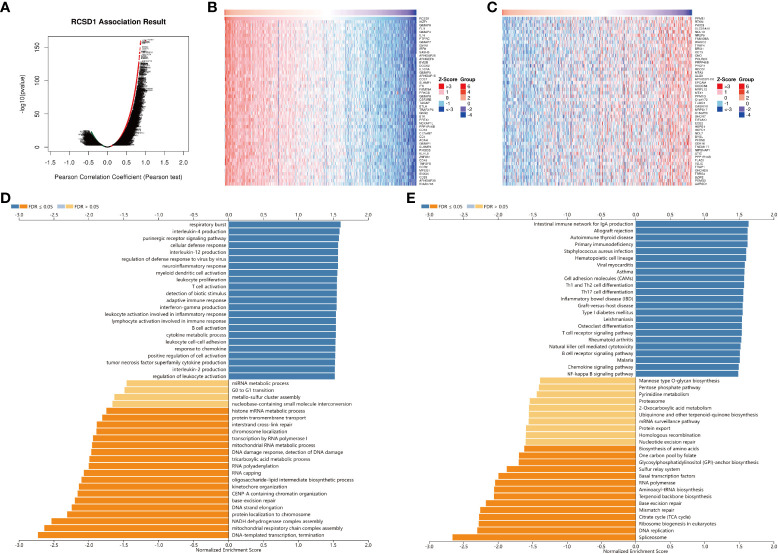
Co-expression genes and enrichment analysis of RCSD1 in LUAD. **(A)** The whole significantly associated genes with RCSD1 distinguished by Pearson test in LUAD cohort. **(B)** Top 50 positive co-expression genes of RCSD1. **(C)** Top 50 negative co-expression genes of RCSD1. **(D)** GO annotations in LUAD. **(E)** KEGG pathways in LUAD.

For gaining the knowledge of RCSD1 biological function in LUAD and LUSC, the LinkedOmics web portal was deployed to check the co-expression patterns of RCSD1. As shown in **Figure 8A** and **Figure S4A**, the genes indicated by red dots were positively correlated with RCSD1, and the green dots were negatively correlated with RCSD1. The **Figures 8B, C** respectively show the heat maps of the top 50 genes positively and negatively associated with RCSD1 in LUAD. IKZF1 (r = 0.8702), GIMAP6 (r = 0.8674) and FLI1 (r = 0.8665) were positively correlated with RCSD1 expression in LUAD (p = 8.15e-160, 1.39e-157, 6.76e-157, respectively) (**Figure 8B**). **Figures S4B, C** showed the heat maps of the top 50 genes positively and negatively correlated with RCSD1 in LUSC, respectively. Then, SASH3 (r=0.9363, p=6.05e-229), EVI2B (r=0.9294, p=3.87e-218) and PTPRC (r=0.9278, p=8.52e-216) are the three genes with the positive correlation with RCSD1 in LUSC (**Figure S4B**). We then performed the GO and KEGG enrichment analyses of the RCSD1-related genes in both LUAD and LUSC cohorts. The results showed that, in LUAD, the RCSD1-related genes were mainly associated with “respiratory burst”, “interleukin-4 production”, “purinergic receptor signaling pathway”, “cellular defense response”, “interleukin-12 production”, “regulation of defense response to virus by virus”, “T cell activation” (**Figure 8D**). In LUSC, the RCSD1-related genes are mainly related to “purinergic receptor signaling pathway”, “regulation of defense response to virus by virus”, “cellular defense response”, “interleukin-10 production” (**Figure S4D**). In addition, the KEGG pathways for RCSD1 and its correlated genes are shown in **Figure 8E** and **Figure S4E**. Among these pathways, many immune-related pathways were highly associated with RCSD1, including “Intestinal immune network for IgA production”, “Allograft rejection”, “Autoimmune thyroid disease”, “Primary immunodeficiency”, and “Th1 and Th2 cell differentiation”. Above, it shows that RCSD1 is closely related with immune infiltration in LUAD and LUSC.

### RCSD1 correlates with infiltration of immune cells in LUAD and LUSC

**Figure 9:**
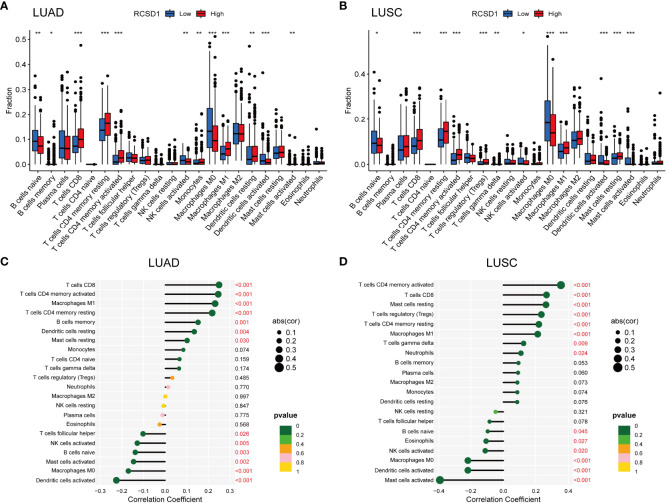
Relationship between RCSD1 and immune cells in LUAD and LUSC. The ratio of 22 immune cells in **(A)** LUAD and **(B)** LUSC tissues in the RCSD1 high and low expression groups. Correlation of RCSD1 and immune cells in **(C)** LUAD and **(D)** LUSC. *, P<0.05; **, P<0.01; ***, P<0.001.

To further assess the effect of RCSD1 on the tumor micro-environment (TME), we first used the CIBERSORT method to analyze the distribution of 22 immune cells in different high and low RCSD1 expression groups. In the LUAD sample, B cells naive (p<0.01), B cells memory (p<0.05), T cells CD8 (p<0.001), T cells CD4 memory resting (p<0.001), T cells CD4 memory activated (p<0.001), NK cells activated (p<0.01), Monocytes (p<0.01), Macrophages M0 (p<0.001), Macrophages M1 (p<0.001), Dentritic cells resting (p<0.01), Dentritic cells activated (p<0.001), and Mast cells activated (p<0.01) was significantly different in the RCSD1 high and low expression group (**Figure 9A**). And in the case of LUSC, RCSD1 low expression group has the higher expression of B cells naive (p<0.05), NK cells activated (p<0.05), Macrophages M0 (p<0.001), Dentritic cells activated (p<0.001), and Mast cells activated (p<0.001) (**Figure 9B**). The expression of T cells CD8 (p<0.001), T cells CD4 memory resting (p<0.001), T cells CD4 memory activated (p<0.001), T cells regulatory (Tregs) (p<0.001), T cells gamma delta (p<0.01), Macrophages M1 (p<0.001) and Mast cells activated (p<0.001) in RCSD1 high expression group was higher than that in RCSD1 low expression group (**Figure 9B**).

We then explored the correlation of immune cells with RCSD1 in both LUAD and LUSC in the R software. In LUAD group, T cells CD8 (p<0.001), T cells CD4 memory activated (p<0.001), Macrophages M1 (p<0.001), T cells CD4 memory resting (p<0.001), B cells memory (p=0.001), Dendritic cells resting (p=0.004), and Mast cells resting (p=0.030) were positively correlated with RCSD1, while T cells follicular helper (p=0.026), NK cells activated (p=0.005), B cells naive (p=0.003), Mast cells activated (p=0.002), Macrophages M0 (p<0.001), and Dentritic cells activated (p<0.001) were negatively correlated with RCSD1 (**Figure 9C**). Similarly, in LUSC group, T cells CD4 memory activated (p<0.001), T cells CD8 (p<0.001), Mast cells resting (p<0.001), T cells regulatory (Tregs) (p<0.001), T cells CD4 memory resting (p<0.001), Macrophages M1 (p<0.001), T cells gamma delta (p=0.009), and Neutrophils (p=0.024) were positively correlated with RCSD1, while B cells naive (p=0.045), Eosinophils (p=0.027), NK cells activated (p=0.020), Macrophages M0 (p<0.001), Dentritic cells activated (p<0.001), and Mast cells activated (p<0.001) were negatively correlated with RCSD1 in LUSC (**Figure 9D** *).*

**Figure 10:**
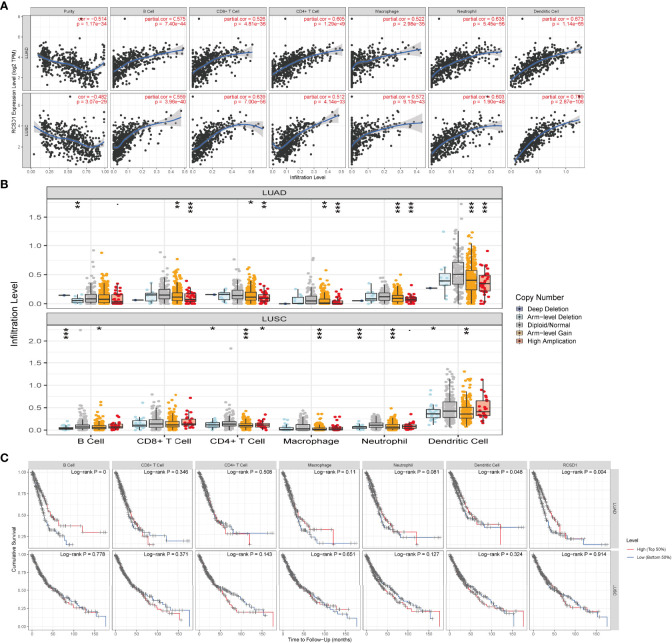
Correlation of RCSD1 expression and immune infiltration level. **(A)** Correlation of RCSD1 with tumor purity and infiltration of different immune cells. **(B)** The infiltration level of each immune subset at different copy numbers of RCSD1 in LUAD and LUSC. **(C)** Effects of multiple tumor immune subsets and RCSD1 expression on the prognosis of LUAD and LUSC patients. *, P<0.05; **, P<0.01; ***, P<0.001.

Immune infiltrating cells in tumor tissues can not only perturb the cytokine signal in tumor micro-environment but also serve a significant part in cancer biology (25). Tumor infiltrating lymphocytes are important predictors for the status of sentinel lymph node and prognosis of cancer patients (26). In our analysis, we explored LUAD and LUSC in TIMER database to determine whether RCSD1 expression was related to the abundance of immune infiltration. Our findings showed that there was a significant positive correlation between RCSD1 expression and immune infiltrates, such as B cells, CD8+ T cells, CD4+ T cells, Macrophages, Neutrophils, and Dendritic cells in LUAD and LUSC. However, RCSD1 expression was negatively associated with tumor purity (**Figure 10A**). **Table 1** shows the correlation analysis of RCSD1 and immune cell biomarkers in LUAD and LUSC based on the TIMER and GEPIA database. In **Table 1**, we can see that RCSD1 has different degrees of correlation with immune cell biomarkers. In both LUAD and LUSC, RCSD1 was significantly associated with all the Gene markers of B cell, CD8+ T Cell, Th1 cell, Th17 cell, Treg cell, T cell exhaustion, M2 Macrophage, TAM, Monocyte, and Natural killer cell (**Table 1**). Moreover, Different copy number alterations of RCSD1 cause changes in the levels of immune cell infiltration. In LUAD group, when arm-level gain and high amplication mutations occur in the RCSD1 gene, it will cause altered infiltration levels of CD8+ T Cell, CD4+ T Cell, Macrophage, Neutrophil, and Dendritic cells (**Figure 10B**). And arm-level deletion and arm-level gain, which occur in RCSD1 of LUSC group, will alter the level of immune infiltration in B cell, CD4+ T Cell, Neutrophil, and Dendritic cells (**Figure 10B**). We next explored the effect of RCSD1 and immune-infiltrating cells on the prognosis of tumor patients. Results showed that increased B cell and Dendritic cells and higher RCSD1 expression indicated the better the prognosis of LUAD patients (**Figure 10C**).

**Table 1:** Correlation analysis between RCSD1 and biomarkers of immune cells in LUAD and LUSC based on the TIMER and GEPIA database.

Description	Gene markers	LUAD						LUSC					
		TIMER				GEPIA		TIMER				GEPIA	
		None		Purity		Tumor		None		Purity		Tumor	
		rho	*P*	rho	*P*	rho	*P*	rho	*P*	rho	*P*	rho	*P*
B cell	CD19	0.62	**4.79e-56**	0.502	**7.45e-33**	0.58	**1.8e-44**	0.692	**1.21e-72**	0.6	**5.09e-48**	0.65	**8.8e-59**
	MS4A1	0.675	**0e+00**	0.565	**7.22e-43**	0.66	**7.3e-62**	0.735	**2.39e-86**	0.663	**8.52e-62**	0.72	**4.9e-78**
	CD79A	0.581	**0e+00**	0.463	**1.46e-27**	0.54	**3.8e-38**	0.693	**7.26e-73**	0.596	**3.60e-47**	0.67	**3.1e-65**
CD8+ T Cell	CD8A	0.663	**0e+00**	0.571	**4.42e-44**	0.65	**2.7e-59**	0.775	**1.19e-101**	0.749	**8.28e-87**	0.76	**1.4e-92**
	CD8B	0.541	**0e+00**	0.454	**2.21e-26**	0.53	**2.7e-36**	0.604	**3.26e-51**	0.604	**8.66e-49**	0.59	**5.7e-47**
	IL2RA	0.675	**7.22e-70**	0.604	**2.38e-50**	0.66	**8.9e-63**	0.792	**3.73e-109**	0.745	**1.98e-85**	0.77	**7.1e-98**
Tfh cell	BCL6	0.146	**8.8e-04**	0.157	**4.78e-04**	0.19	**3.3e-05**	-0.015	7.4e-01	0.049	2.88e-01	-0.0057	0.9
	CXCR5	0.68	**0e+00**	0.575	**1.09e-44**	0.17	**0.00016**	0.779	**2.88e-103**	0.717	**1.42e-76**	0.18	**7.9e-05**
	ICOS	0.753	**3.07e-95**	0.665	**3.68e-64**	0.74	**8.1e-86**	0.837	**4.6e-133**	0.79	**5.29e-103**	0.82	**8.2e-121**
Th1 cell	IL12RB1	0.813	**1.16e-122**	0.752	**8.63e-91**	0.81	**2.2e-112**	0.898	**1.51e-180**	0.871	**8.41e-149**	0.89	**2.8e-164**
	STAT1	0.464	**0e+00**	0.378	**3.77e-18**	0.45	**2.4e-25**	0.522	**2.33e-36**	0.493	**1.57e-30**	0.49	**4e-30**
	CCR5	0.811	**0e+00**	0.749	**9.07e-90**	0.8	**4.4e-111**	0.887	**2.12e-169**	0.858	**2.93e-139**	0.88	**2.9e-156**
Th2 cell	CCR4	0.769	**0e+00**	0.721	**2.90e-80**	0.78	**3.2e-99**	0.875	**1.3e-159**	0.84	**4.08e-128**	0.88	**5.8e-157**
	STAT6	0.227	**2.03e-07**	0.293	**3.04e-11**	0.24	**7.1e-08**	0.156	**4.63e-04**	0.18	**7.82e-05**	0.11	**0.013**
	HAVCR1	0.042	3.46e-01	0.061	1.76e-01	0.093	**0.04**	0.368	**1.66e-17**	0.378	**1.22e-17**	0.35	**3.9e-15**
Th17 cell	IL21R	0.754	**0e+00**	0.67	**1.67e-65**	0.75	**4.5e-88**	0.855	**3.15e-144**	0.813	**1.24e-113**	0.84	**8.6e-131**
	IL23R	0.44	**9.02e-26**	0.42	**1.87e-22**	0.47	**2e-28**	0.404	**4.27e-21**	0.336	**4.74e-14**	0.37	**2e-17**
	CCR6	0.717	**0e+00**	0.641	**2.03e-58**	0.71	**7.1e-75**	0.815	**1.51e-120**	0.746	**7.90e-86**	0.8	**5.4e-109**
Treg cell	STAT5B	0.522	**2.47e-37**	0.559	**6.72e-52**	0.47	**4.4e-28**	0.271	**7.49e-10**	0.321	**7.23e-13**	0.19	**1.7e-05**
	NT5E	0.159	**2.95e-04**	0.143	**1.51e-03**	0.15	**0.00078**	0.217	**9.27e-07**	0.118	**9.82e-03**	0.2	**8.6e-06**
	CCR8	0.707	**2.34e-79**	0.633	**1.22e-56**	0.7	**1.3e-71**	0.791	**1.25e-108**	0.743	**1.01e-84**	0.77	**8.4e-97**
	IL7R	0.793	**0e+00**	0.733	**3.25e-84**	0.8	**2.3e-108**	0.668	**5.57e-66**	0.573	**6.18e-43**	0.65	**2.3e-59**
T cell exhaustion	PDCD1	0.629	**4.93e-58**	0.532	**2.04e-37**	0.62	**5.5e-52**	0.791	**1.9e-108**	0.748	**1.22e-86**	0.78	**1.1e-99**
	CTLA4	0.681	**0e+00**	0.576	**6.51e-45**	0.68	**9.1e-66**	0.795	**2.23e-110**	0.737	**6.27e-83**	0.79	**1.4e-109**
	LAG3	0.506	**0e+00**	0.402	**1.50e-20**	0.47	**3.1e-28**	0.665	**3e-65**	0.63	**4.15e-54**	0.61	**8e-51**
	GZMB	0.435	**3.56e-25**	0.303	**5.94e-12**	0.39	**2.4e-19**	0.644	**5.23e-60**	0.571	**1.51e-42**	0.61	**5e-50**
M1 Macrophage	NOS2	0.315	**2.46e-13**	0.246	**3.30e-08**	0.34	**3.8e-14**	0.115	**1e-02**	0.148	**1.17e-03**	0.098	**0.031**
	IRF5	0.451	**0e+00**	0.363	**8.37e-17**	0.43	**6.7e-23**	0.214	**1.31e-06**	0.193	**2.15e-05**	0.17	**0.00022**
	PTGS2	-0.099	**2.43e-02**	-0.115	**1.09e-02**	-0.1	**0.023**	0.047	0.296	-0.048	2.96e-01	0.045	0.32
M2 Macrophage	CD163	0.666	**0e+00**	0.604	**1.94e-50**	0.62	**2.6e-52**	0.78	**9.63e-104**	0.73	**1.10e-80**	0.76	**1.1e-92**
	MRC1	0.611	**0e+00**	0.557	**1.63e-41**	0.62	**6.1e-53**	0.703	**9.08e-76**	0.642	**1.14e-56**	0.69	**2.6e-70**
	CD209	0.625	**0e+00**	0.551	**1.58e-40**	0.63	**3.3e-54**	0.634	**1.25e-57**	0.571	**1.11e-42**	0.62	**5.2e-53**
	MS4A4A	0.672	**0e+00**	0.6	**1.31e-49**	0.68	**4.4e-66**	0.793	**2.1e-109**	0.743	**5.48e-85**	0.78	**7.4e-100**
TAM	CCL2	0.374	**0e+00**	0.253	**1.26e-08**	0.37	**5.1e-17**	0.57	**1.38e-44**	0.501	**1.26e-31**	0.55	**8.2e-40**
	CD68	0.599	**0e+00**	0.528	**8.61e-37**	0.59	**1.1e-46**	0.665	**2.44e-65**	0.586	**2.32e-45**	0.62	**2.2e-52**
	IL10	0.615	**6.19e-55**	0.518	**3.08e-35**	0.6	**3.6e-49**	0.644	**4.31e-60**	0.589	**7.11e-46**	0.63	**5.6e-56**
Monocyte	CD14	0.573	**0e+00**	0.484	**2.81e-30**	0.57	**9.3e-43**	0.76	**1.29e-95**	0.681	**2.32e-66**	0.74	**7.9e-85**
	CD33	0.657	**0e+00**	0.582	**4.54e-46**	0.63	**1.2e-54**	0.809	**2.29e-117**	0.755	**4.63e-89**	0.78	**1.2e-102**
	ITGAX	0.656	**1.08e-64**	0.569	**1.49e-43**	0.62	**1.6e-52**	0.777	**3.18e-102**	0.703	**2.56e-72**	0.71	**6.6e-76**
Natural killer cell	B3GAT1	0.259	**2.31e-09**	0.23	**2.51e-07**	0.28	**4.3e-10**	0.58	**1.87e-46**	0.545	**3.28e-38**	0.58	**1.5e-44**
	KIR3DL1	0.286	**3.49e-11**	0.226	**3.79e-07**	0.32	**9.2e-13**	0.486	**4.96e-31**	0.45	**3.25e-25**	0.5	**4e-32**
	CD7	0.484	**0e+00**	0.375	**6.61e-18**	0.47	**2.6e-27**	0.755	**2.03e-93**	0.688	**3.17e-68**	0.74	**6e-86**
Neutrophils	FCGR3A	0.601	**0e+00**	0.523	**5.15e-36**	0.6	**2.3e-48**	0.785	**8.73e-106**	0.74	**5.81e-84**	0.77	**1.5e-95**
	CCR7	0.755	**0e+00**	0.669	**2.33e-65**	0.75	**2.9e-89**	0.802	**8.83e-114**	0.747	**2.44e-86**	0.79	**3e-106**
	CD55	-0.081	6.66e-02	-0.063	1.65e-01	-0.068	0.13	0.242	**4.05e-08**	0.176	**1.09e-04**	0.21	**4.2e-06**
	ITGAM	0.632	**0e+00**	0.565	**6.15e-43**	0.65	**4.6e-59**	0.721	**1.5e-81**	0.652	**4.95e-59**	0.72	**3.2e-78**
Dendritic cell	CD1C	0.467	**2.77e-29**	0.394	**8.39e-20**	0.47	**2.4e-27**	0.61	**2.61e-52**	0.475	**3.29e-28**	0.59	**3e-47**
	THBD	0.395	**1.1e-20**	0.317	**6.08e-13**	0.43	**3.8e-23**	-0.021	6.36e-01	-0.089	5.09e-02	-0.012	0.8
	NRP1	0.281	**1.01e-10**	0.268	**1.48e-09**	0.28	**3.6e-10**	0.531	**9.68e-38**	0.444	**1.70e-24**	0.49	**7.2e-31**

Bold values indicate P < 0.05.

### Relationship between RCSD1 and immune molecules in lung cancer

**Figure 11:**
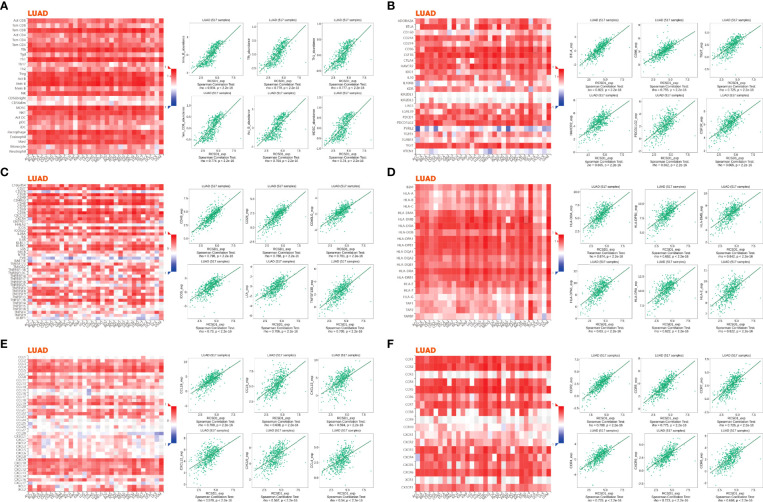
Associations of the RCSD1 expression level with lymphocytes, immunomodulators and chemokines in LUAD. **(A)** Correlations between abundance of tumor-infiltrating lymphocytes (TILs) and RCSD1 (plus the six TILs with the highest correlation). **(B–D)** Correlations between immunomodulators and RCSD1 (plus the six immunomodulators with the highest correlation, respectively). **(E, F)** Correlations between chemokines (or receptors) and RCSD1 [plus the six chemokines (or receptors) with the highest correlation, respectively].

Next, we investigated the connections between RCSD1 expression and various immune signatures to broaden the cognition of the correlation between RCSD1 and immune infiltration. **Figure 11A** shows the relationship between the abundance of TIL and the expression of RCSD1. Among them, Immature B cell, T follicular helper cell, and Th1 have the highly correlation with RCSD1 in LUAD (**Figure 11A**). **Figures 11B, C** show the correlation between RCSD1 and the markers of immunoinhibitor and immunostimulator, separately. And among the immunoinhibitor factors, RCSD1 has the high correlation with BTLA, CD96, and TIGIT (**Figure 11B**). In these immunostimulator factors, RCSD1 showed the high correlation with CD48, CD28, and CD40LG (**Figure 11C**). The MHC molecules, such as HLA-DOA, HLA-DPB1, and HLA-DMB, showed the high correlation with RCSD1 (**Figure 11D**). In the chemokine (receptor) tab, we can examine the chemokines (or receptors) that may be regulated by RCSD1. It can now be seen that, in the LUAD, the chemokine highly associated with RCSD1 are the CCL19, CCL5, CXCL13, CXCL12, CXCL9, CCL4 (**Figure 11E**), and the receptors highly associated with RCSD1 are the CCR2, CCR5, CCR7, CXCR6, CCR6 (**Figure 11F**). **Figure S5** demonstrated the immunological features associated with RCSD1 in LUSC. **Figure S5A** showed the significant correlation of RCSD1 with Imm _B, Tfh, and Myeloid derived suppressor cell (MDSC). **Figures S5B, C** show the correlation between RCSD1 and the marker factors of immunoinhibitor and immunostimulator, respectively. And among the immunoinhibitor factors, RCSD1 has the high correlation with HAVCR2, CD96, and CSF1R (**Figure S5B**). In LUSC, RCSD1 showed the high correlation with CD28, CD48, and CD40LG among the immunostimulator factors (**Figure S5C**). The MHC molecules, such as HLA-DPA1, HLA-DPB1, and HLA-DMB, showed the high correlation with RCSD1 in LUSC (**Figure S5D**). It can also be seen that, in the LUSC, the chemokine highly associated with RCSD1 are the CCL19, CCL4, CCL5 (**Figure S5E**), and the receptors highly associated with RCSD1 are the CCR2, CCR5, CCR4 (**Figure S5F**). Thus, we confirmed that RCSD1 is widely involved in regulating multiple immune molecules in LUAD and LUSC, thereby affecting immune infiltration in the tumor micro-environment.

### Analysis of RCSD1 versus immunotherapy

**Figure 12:**
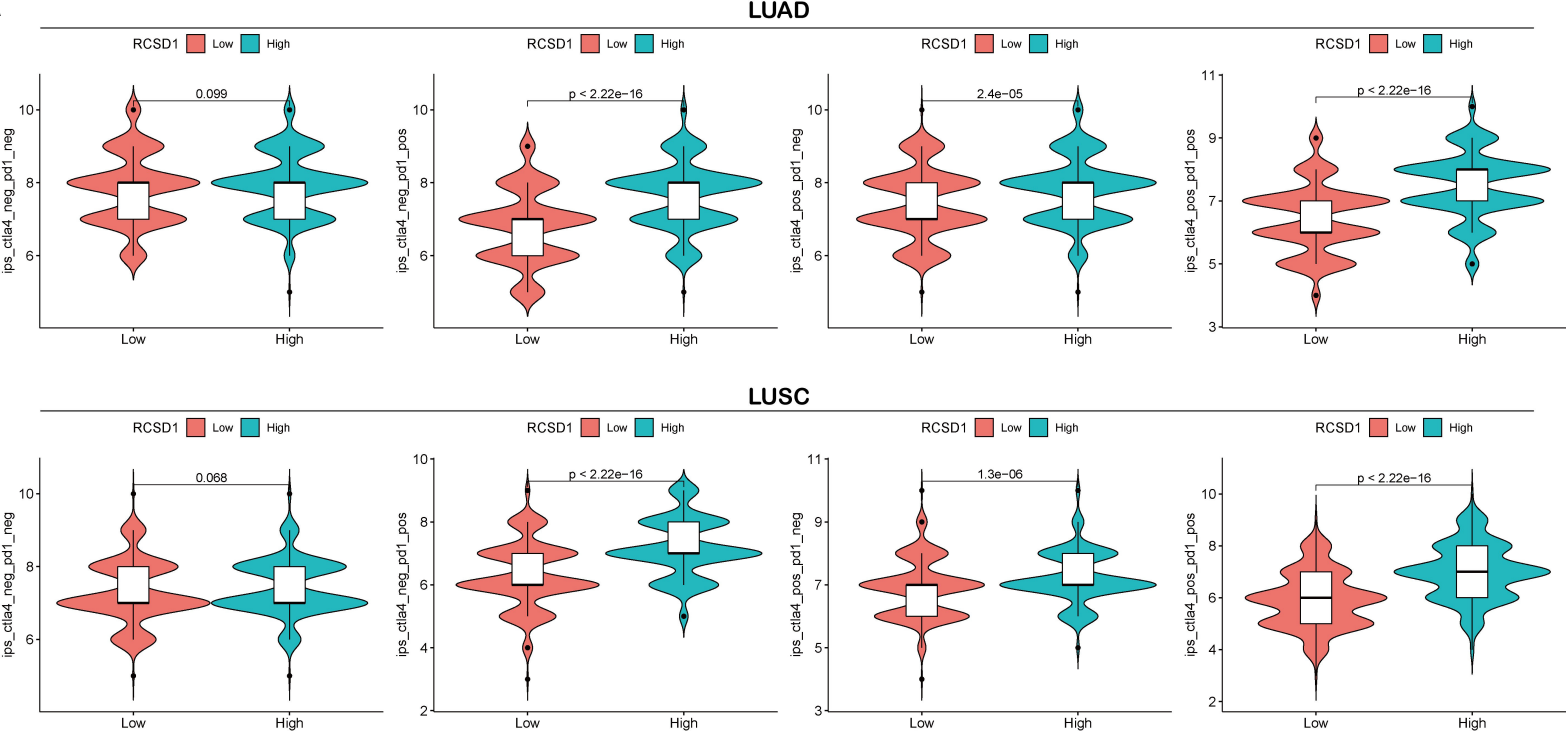
Relationship between RCSD1 expression and immunotherapy. **(A)** IPS score for the high and low RCSD1 expression group in LUAD. **(B)** IPS score for the high and low RCSD1 expression group in LUSC.

Cytotoxic T-lymphocyte-associated antigens 4 (CTLA-4) and programmed death 1 (PD-1) immune checkpoints are negative regulators of T-cell immune function. The PD-L1 inhibitors and the CTLA-4 inhibitors increase the average life expectancy of cancer patients by passing through different cellular immune pathways (27–29). IPS can determine the tumor immunogenicity and predict the response of multiple tumor types to treatment with immune checkpoint inhibitors (30). The higher the IPS, the better the patient is receiving immunotherapy. Through the analysis of immunotherapy in the TICA database, we can observe the effect of immunotherapy between the high and low RCSD1 expression groups. The abscissa of the violin plot represents the grouping of the RCSD1, and we divided the RCSD1 into two groups of high and low expression groups, and the vertical coordinate represents the IPS (**Figure 12**). In the PD1 negative/CTLA4 negative group, RCSD1 expression has no significance for immunotherapy. In the PD1 positive/CTLA4 negative, PD1 negative/CTLA4 positive, PD1 positive/CTLA4 positive groups, the higher the RCSD1 expression, the better the effect of receiving immunotherapy (**Figure 12**). These results suggest that patients in the high RCSD1 expression group may show a better response to immunotherapy.

## Discussion

The RCSD1 gene encodes a highly phosphorylation-dependent cytoskeletal regulatory molecule and participates in important components of mitosis (31). However, when the RCSD1 gene is dysregulated, such as a gene fusion occurs, the cell’s cytoskeleton regulation capacity will be altered, affecting cell function (9, 10, 32). Currently, the mechanism of RCSD1 in the development of other human malignancies has not been further explored. Therefore, we investigated the mRNA and protein expression of RCSD1 in pan-cancer through public databases. The relationship between RCSD1 and patient prognosis, clinical data, gene mutations, tumor immune infiltration, tumor immune markers, and immunotherapy provides new insights for future clinical diagnosis and treatment.

There may be several reasons for the influence of RCSD1 on the prognosis of tumor patients. Firstly, through the Timer database, we found that the RCSD1 expression was lower in BLCA, BRCA, LUAD, LUSC, COAD, KICH, KIRP, PRAD, READ, STAD, THCA, and UCEC, while it was higher in a few tumors (CHOL, HNSC, KIRC, and so on). Meanwhile, we performed a complementary analysis of the Timer database using the GEPIA database, and the expression of RCSD1 in 15 human tumors was lower than that in normal tissues, which was consistent with the conclusion in the TIMER database. Our analysis of RCSD1 total protein and phosphorylated proteins in pan-cancer using CPTAC database revealed that RCSD1 total protein expression was lower in COAD, LIHC, and LUAD tissues than in normal tissues. However, the total protein expression of RCSD1 in GBM, PAAD, KIRC, and UCEC was higher than that in normal tissues. Previous studies have shown that CapZIP, encoded by the RCSD1 gene, is a substrate of SAPKs (stress-activated protein kinases). Stress-activated members of the mitogen-activated protein kinase family phosphorylate CapZIP at many sites, including Ser-68, Ser-83, Ser-108, and Ser-216 (7). In our study, RCSD1 (NP_443094.3) was found to be phosphorylated at multiple sites of the pan-cancer. In LUAD and BRCA cohorts, the RCSD1 phosphoprotein expression at all sites was lower than in the corresponding normal tissues. However, in KIRC, RCSD1 phosphorylated proteins at S68, S105, S105S108, and S284 showed lower expression in tumors than in normal tissues; the RCSD1 expression of phosphoproteins at S116S120, S216, S267S268, and S298 was higher than that in normal tissues. This finding can help the subsequent basic experiments to deeply explore the specific site and mechanism of RCSD1 phosphorylated protein in the mechanism of tumor development and development. Meanwhile, after a comprehensive analysis of the GEPIA database and the Kaplan-Meier survival curves, we found that the low expression of RCSD1 in most tumors (HNSC, KIRC, LUAD, THYM, SARC, and so on) was associated with poor patient prognosis (OS) in tumors. However, the high expression of RCSD1 in only a few tumors (LGG, UVM, TGCT, and ESCC) was associated with poor tumor patient prognosis (OS). Above, the mRNA expression and the protein expression level of the RCSD1 in the different tumors may be the important factors affecting the patient prognosis.

Through previous studies, we learned that genetic mutations in RCSD1 may induce hematological malignancies (33), so here, we investigated the type and status of mutation and mutations that may occur in the RCSD1 gene in pan-cancer, and the effect of the RCSD1 gene mutation on patient prognosis. We first analyzed the gene mutation frequency, mutation types, mutation site of RCSD1 in pan-cancer by using the cBioportal web site, RCSD1 mutation rates were found to be high in CHOL, BLCA, LIHC, BRCA, LUAD, SARC, and LUSC. And most types were the Amplification. We then analyzed the effect of genetic mutations in RCSD1 on patient prognosis, found that the RCSD1 genetic alteration significantly shortened the DFS in COAD patients and the OS in PCPG patients. The above results indicate that genetic mutations of RCSD1 in tumors are potential factors that induce poor prognosis in patients.

In parallel, we retrieved 50 proteins that showed interactions with RCSD1 in pan-cancer by the String database. Among these genes that interact with RCSD1 include USP7, CD2AP, and CAPZA1. Previous studies have shown that USP7 is able to act synergistically with PI3K inhibitors to inhibit breast cancer development (34). The CD2AP was able to inhibit the metastasis of gastric cancer (35). CAPZA1 is lowly expressed in tumors, when CAPZA1 is phosphorylated, the adhesion and migration of PRAD, PAAD, and LIHC cells increase, leading to poor patient prognosis (36–38). At the same time, we also screened the 100 genes with the strong correlation with RCSD1 in the GEPIA database, in which IKZF1 (r=0.87) and DOCK2 (r=0.87) were highly positively associated with RCSD1. Studies have shown that genetic mutations in IKZF1 are associated with poor prognosis in hematologic tumors and colorectal tumors (39–41); Low expression of DOCK2 is associated with a poor prognosis in colorectal cancer and lung cancer patients (42–44). Through GO and KEGG enrichment analysis, we can see that RCSD1-related genes are highly correlated with cellular immunomodulatory processes such as “T cell activation”, “lymphocyte differentiation”, and “mononuclear cell proliferation”. Moreover, it is highly enriched in the immune-related signaling pathways such as “chemokine signaling pathway”, “VEGF signaling pathway”, and “T cell receptor signaling pathway”. The above analysis proved that RCSD1 and its related genes are closely related to the process of tumorigenesis, and that the expression of RCSD1 may affect the prognosis of patients. At the same time, the expression of RCSD1 was significantly different in most of the tumor immune subtypes and tumor immune molecular subtypes, which suggested that RCSD1 may be involved in regulating tumor immune related processes, thereby affecting patient prognosis.

In addition, we selected LUAD and LUSC focusing on the correlation of RCSD1 and patient clinical data, found that RCSD1 was associated with individual tumor stage, patient age, smoking history, and lymph node metastasis in LUAD patients. RCSD1 is also associated with individual tumor stage, histological subtypes, and TP53 mutational status in LUSC patients. In LUAD, we then explored the 50 genes with a positive and negative correlation with RCSD1. In the LUAD group, IKZF1 and GIMAP6 showed the strong positive correlation with RCSD1. IKZF1 is a known tumor-suppressor gene (45, 46), and high GIMAP6 expression level is also associated with a favorable prognosis in patients with LUAD and LIHC (47, 48). In LUSC, SASH3 showed the strong positive association with RCSD1, and SASH3 is currently known to be a LUAD suppressor (49). The above results indicate that RCSD1 is potentially associated with lung cancer prognosis, but the specific occurrence mechanism remains unclear. Therefore, we further explored the relationship between RCSD1 and the immune mechanisms of lung cancer. The results showed that RCSD1 was correlated with immune cells in lung cancer, moreover, increased tumor immune-infiltrating cells and increased RCSD1 expression were associated with better prognosis of lung cancer patients. There was a significant correlation between RCSD1 and most TIL, and immunotherapy with high RCSD1 expression was better. The above results indicate that RCSD1 mainly regulates tumor-related immune mechanisms, involved in the development and development of lung cancer and affects patient prognosis.

This study improves our understanding of the relationship between RCSD1 and pan-cancer, but several limitations remain. First, we mainly explored the analysis of RCSD1 expression and immune related processes in pan-cancer. However, the biological process and molecular mechanism of RCSD1 in tumor progression need more basic experimental studies. Second, we only investigated the expression of RCSD1 phosphorylated proteins, but the specific phosphorylation mechanism has not been deeply explored.

Taken together, our results demonstrate that RCSD1 is significantly differentially expressed in different human tumors and that its expression level correlates with clinical case characteristics and prognosis of pan-cancer patients. The expression of RCSD1 is closely related to the immune infiltration of lung cancer cells, and RCSD1 may partly affect the prognosis by regulating the immune infiltration in lung cancer patients. RCSD1 may serve as a prognostic biomarker for the prognosis associated with immune infiltration in lung cancer. In conclusion, we conducted a comprehensive assessment of RCSD1, revealing its potential role as an indicator of patient prognosis and its immunoregulation effect.

Therefore, in future clinical work, the detection of RCSD1 expression levels in cancer patients could be used to evaluate the disease and predict the prognosis of patients. We look forward to further studies of RCSD1 to progressively elucidate the biological role of RCSD1 in the tumor immune micro-environment and prognosis.

## Data availability statement

The original contributions presented in the study are included in the article/**Supplementary Material**. Further inquiries can be directed to the corresponding author.

## Author contributions

Conception and design: All authors. Administrative support: HQ, HT. Provision of study materials or patients: HQ, HY, HT. Collection and assembly of data: All authors. Data analysis and interpretation: All authors. All authors contributed to the article and approved the submitted version.

## Funding

This work was supported by Shandong Branch of National Clinical Research Center for Respiratory Diseases (Funding number:21-1-2-3-zyyd-nsh).

## Conflict of interest

The authors declare that the research was conducted in the absence of any commercial or financial relationships that could be construed as a potential conflict of interest.

## Publisher’s note

All claims expressed in this article are solely those of the authors and do not necessarily represent those of their affiliated organizations, or those of the publisher, the editors and the reviewers. Any product that may be evaluated in this article, or claim that may be made by its manufacturer, is not guaranteed or endorsed by the publisher.

## Supplementary material

Supplementary Figure 1 RCSD1 phosphoprotein expression in tumors. **(A–G)** in LUAD. **(H–L)** in BRCA. **(M–T)** in KIRC.

Supplementary Figure 2 The relationship between RCSD1 expression and pan-cancer molecular subtypes. **(A)** in ACC, **(B)** in BRCA, **(C)** in COAD, **(D)** in ESCA, **(E)** in HNSC, **(F)** in KIRP, **(G)** in LGG, **(H)** in LIHC, **(I)** in LUSC, **(J)** in OV, **(K)** in PCPG, **(L)** in PRAD, **(M)** in STAD, **(N)** in UCEC.

Supplementary Figure 3 RCSD1 differential expression in LUSC with different clinical subgroups. On **(A)** sample types, **(B)** individual cancer stages, **(C)** patient’s race, **(D)** patient’s gender, **(E)** patient’s age, **(F)** patient’s smoking habits, **(G)** histological subtypes, **(H)** nodal metastasis status and **(I)** TP53 mutation status. *, P < 0.05; **, P < 0.01; ***, P < 0.001.

Supplementary Figure 4 Co-expression genes and enrichment analysis of RCSD1 in LUSC. **(A)** The whole significantly associated genes with RCSD1 distinguished by Pearson test in LUSC cohort. **(B)** Top 50 positive co-expression genes of RCSD1. **(C)** Top 50 negative co-expression genes of RCSD1. **(D)** GO annotations of RCSD1. **(E)** KEGG pathways of RCSD1.

Supplementary Figure 5 Associations of the RCSD1 expression level with lymphocytes, immunomodulators and chemokines in LUSC. **(A)** Correlations between abundance of tumor-infiltrating lymphocytes (TILs) and RCSD1 (plus the six TILs with the highest correlation). **(B–D)** Correlations between immunomodulators and RCSD1 (plus the six immunomodulators with the highest correlation, respectively). **(E, F)** Correlations between chemokines (or receptors) and RCSD1 [plus the six chemokines (or receptors) with the highest correlation, respectively].

Supplementary Table 1 The relationship between RCSD1 and lymphocytes.

Supplementary Table 2 The relationship between RCSD1 and Immunoinhibitors.

Supplementary Table 3 The relationship between RCSD1 and immunostimulators.

Supplementary Table 4 The relationship between RCSD1 and MHC molecules.

Supplementary Table 5 The relationship between RCSD1 and chemokines.

Supplementary Table 6 The relationship between RCSD1 and receptors.
