# Supplementary figures and images for: Pan-cancer analysis reveals the relationship between RCSD1 immune infiltration and clinical prognosis in human tumors
Source: Front Immunol. 2022 Oct 13;13:1008778. doi: 10.3389/fimmu.2022.1008778 (PMC9606721; doi:10.3389/fimmu.2022.1008778)

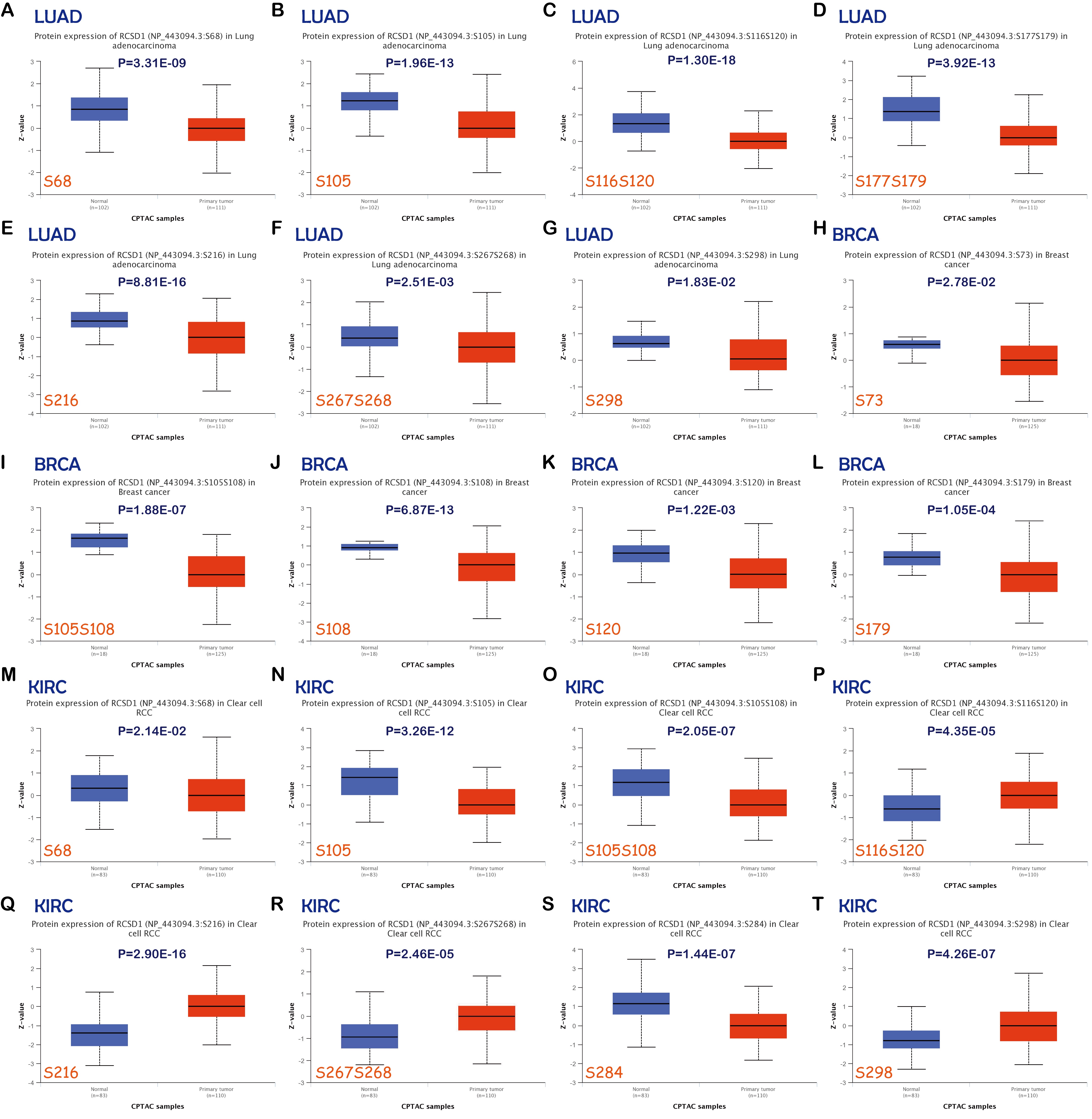

Supplement: Supplementary Figure 1 — RCSD1 phosphoprotein expression in tumors. (A–G) in LUAD. (H–L) in BRCA. (M–T) in KIRC. [file Image_1.jpeg]

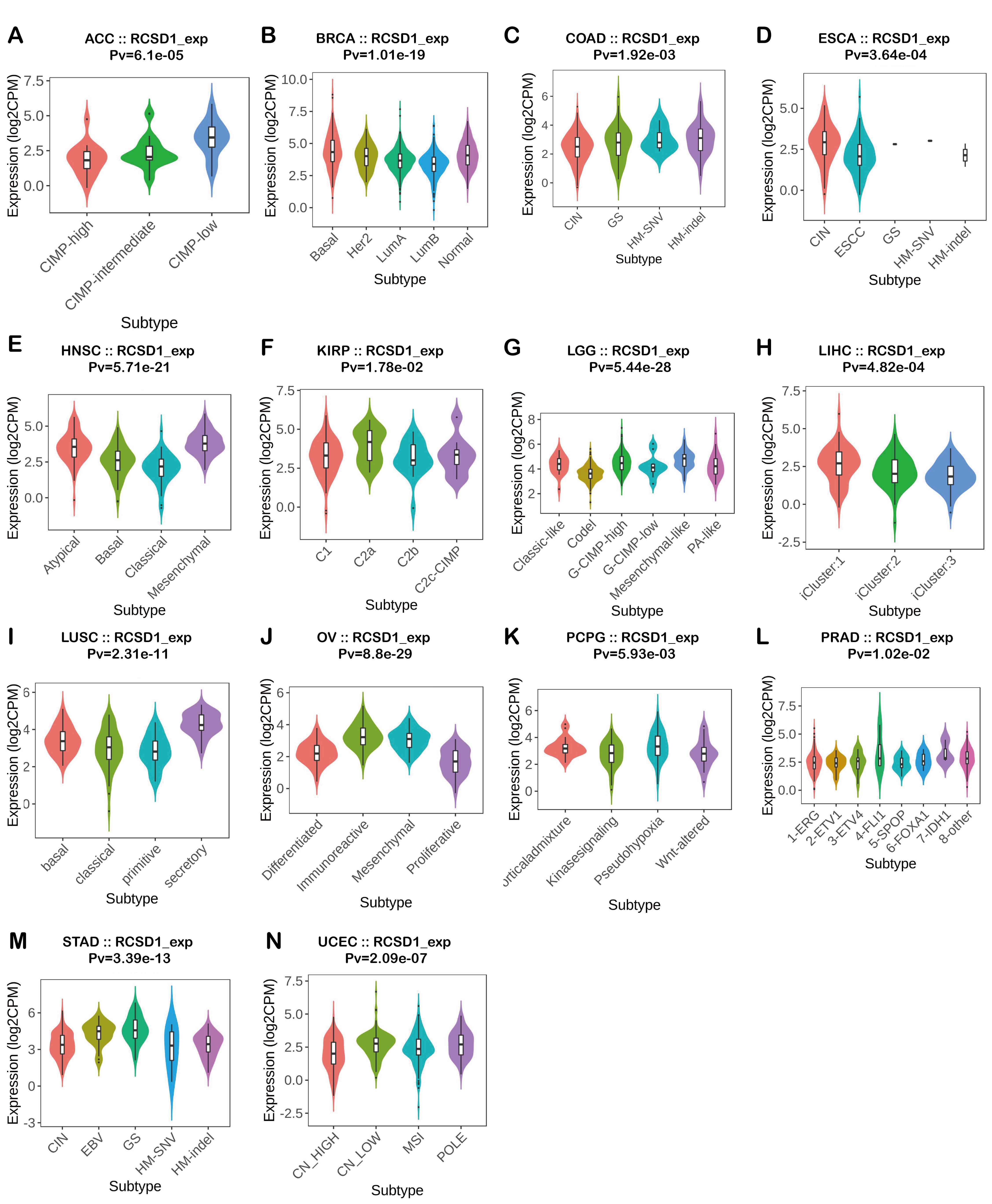

Supplement: Supplementary Figure 2 — The relationship between RCSD1 expression and pan-cancer molecular subtypes. (A) in ACC, (B) in BRCA, (C) in COAD, (D) in ESCA, (E) in HNSC, (F) in KIRP, (G) in LGG, (H) in LIHC, (I) in LUSC, (J) in OV, (K) in PCPG, (L) in PRAD, (M) in STAD, (N) in UCEC. [file Image_2.jpeg]

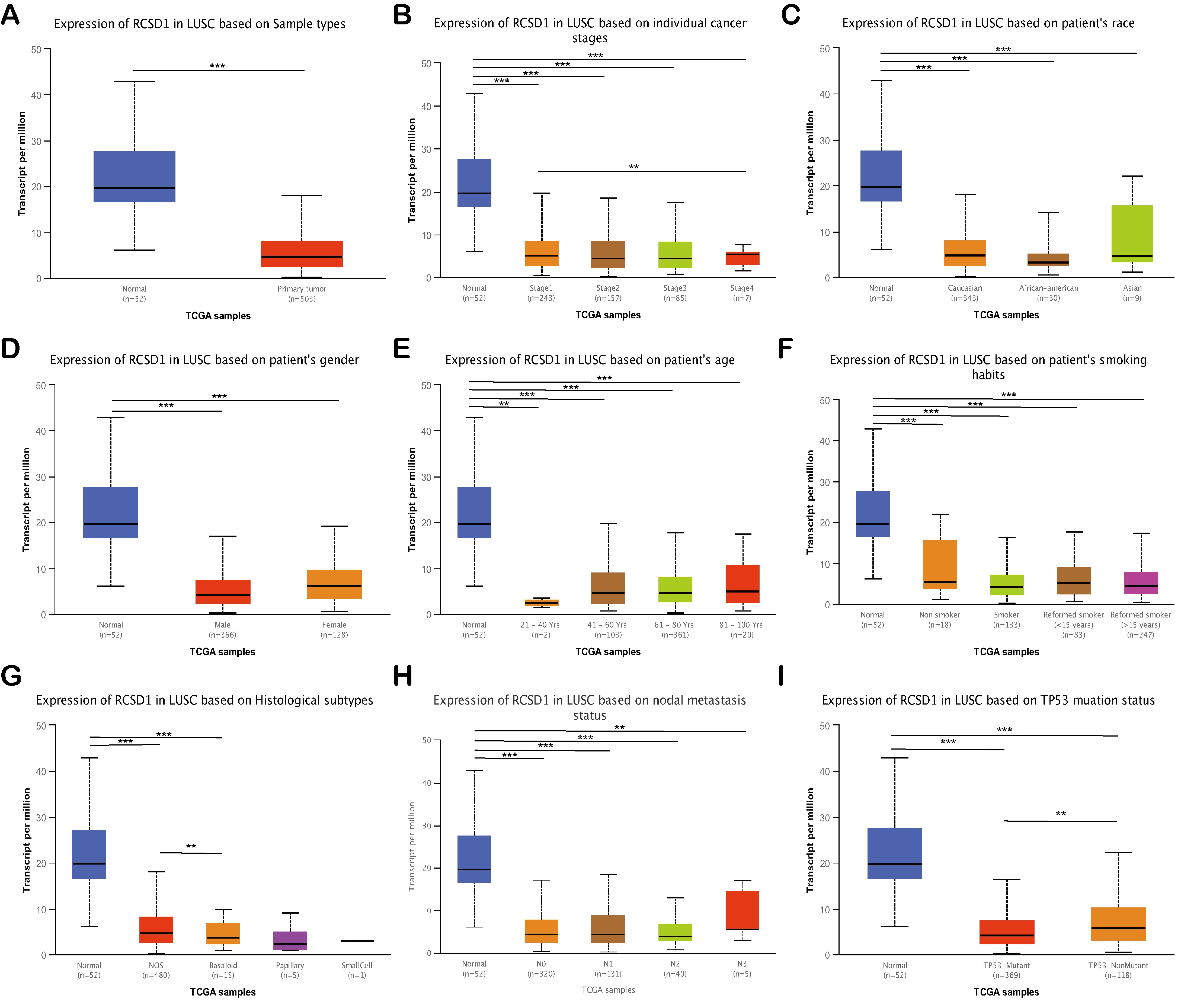

Supplement: Supplementary Figure 3 — RCSD1 differential expression in LUSC with different clinical subgroups. On (A) sample types, (B) individual cancer stages, (C) patient’s race, (D) patient’s gender, (E) patient’s age, (F) patient’s smoking habits, (G) histological subtypes, (H) nodal metastasis status and (I) TP53 mutation status. *, P < 0.05; **, P < 0.01; ***, P < 0.001. [file Image_3.jpeg]

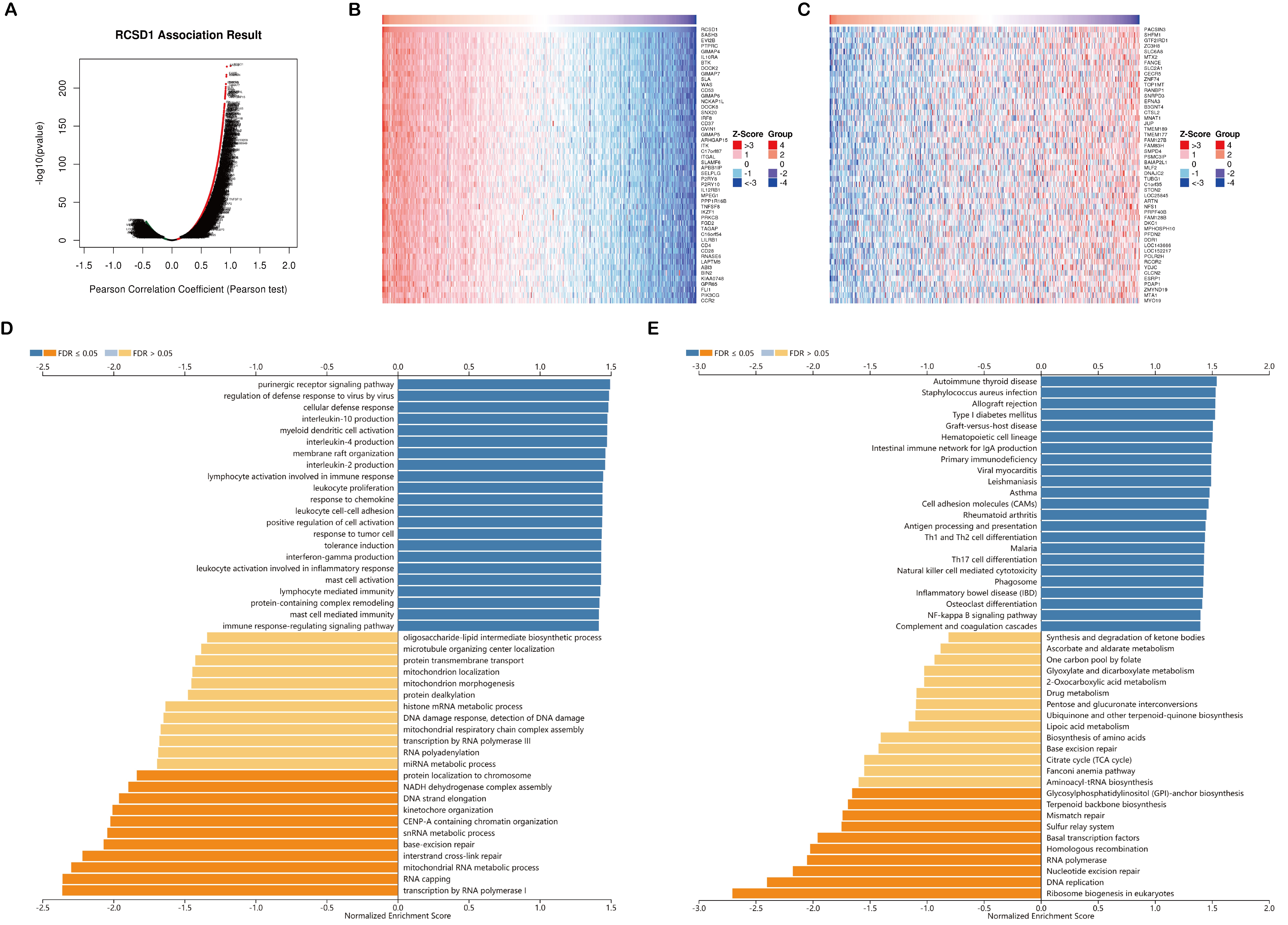

Supplement: Supplementary Figure 4 — Co-expression genes and enrichment analysis of RCSD1 in LUSC. (A) The whole significantly associated genes with RCSD1 distinguished by Pearson test in LUSC cohort. (B) Top 50 positive co-expression genes of RCSD1. (C) Top 50 negative co-expression genes of RCSD1. (D) GO annotations of RCSD1. (E) KEGG pathways of RCSD1. [file Image_4.jpeg]

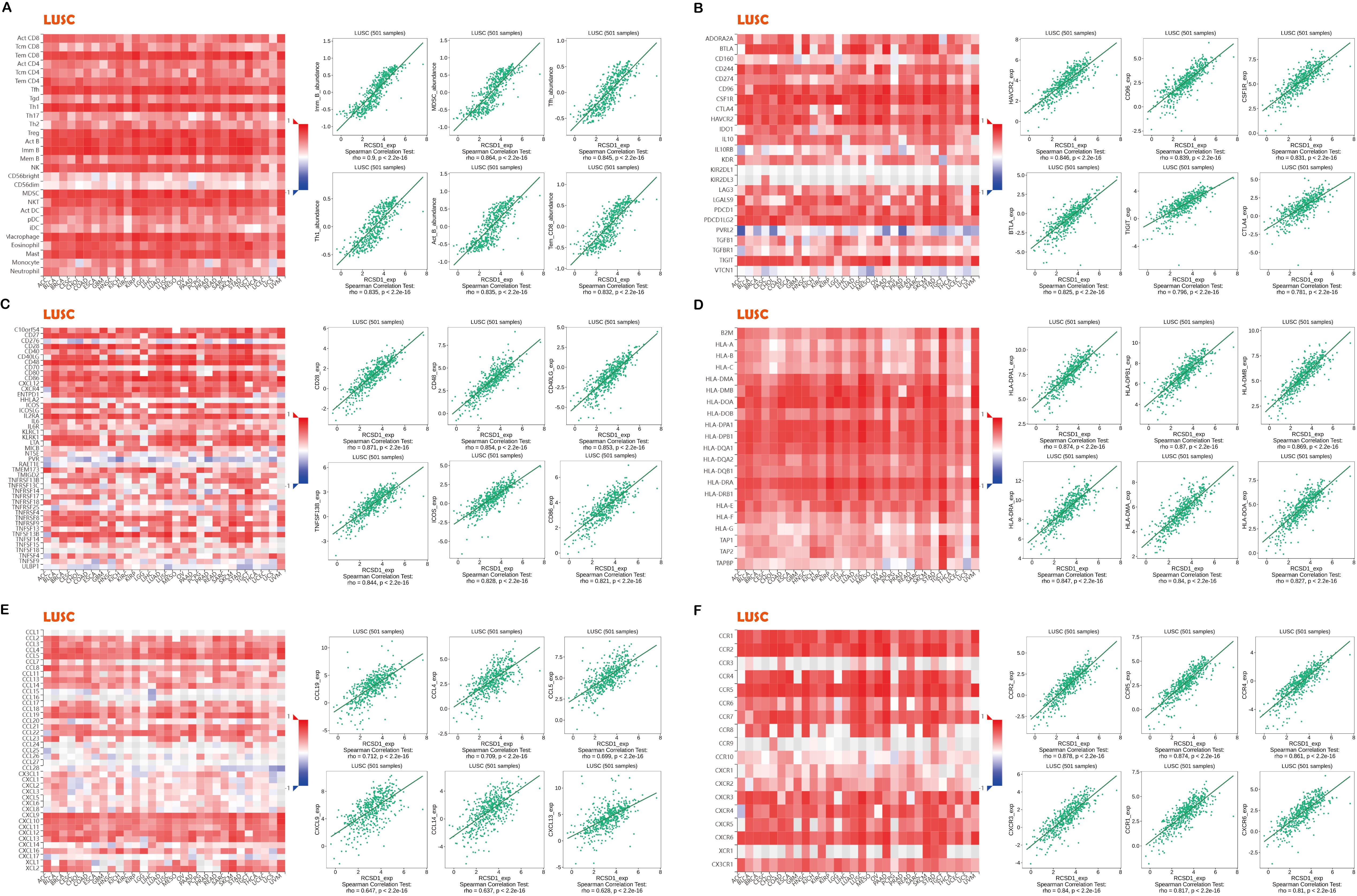

Supplement: Supplementary Figure 5 — Associations of the RCSD1 expression level with lymphocytes, immunomodulators and chemokines in LUSC. (A) Correlations between abundance of tumor-infiltrating lymphocytes (TILs) and RCSD1 (plus the six TILs with the highest correlation). (B–D) Correlations between immunomodulators and RCSD1 (plus the six immunomodulators with the highest correlation, respectively). (E, F) Correlations between chemokines (or receptors) and RCSD1 [plus the six chemokines (or receptors) with the highest correlation, respectively]. [file Image_5.jpeg]
